# The role of a synanthropic bird in the nest niche expansion of a secondary cavity nester to man‐made structures

**DOI:** 10.1002/ece3.9188

**Published:** 2022-08-04

**Authors:** Jing‐Chia Guo, Jo‐Szu Tsai, Jhih‐Syuan Wang, Ya‐Wen Lin, Pei‐Jen Lee Shaner, Chih‐Ming Hung

**Affiliations:** ^1^ Department of Life Sciences National Taiwan Normal University Taipei Taiwan; ^2^ Department of Biological Resources National Chiayi University Chiayi Taiwan; ^3^ Biodiversity Research Center Academia Sinica Taipei Taiwan

**Keywords:** Asian house martin, facilitation, russet sparrow, secondary cavity nester

## Abstract

Species with similar ecological characters often compete with each other; however, a species may also facilitate the survival or reproduction of another ecologically similar species, although such interaction is rarely documented in birds. Here, we reported a facilitative species interaction between Asian house martins (*Delichon dasypus*) and russet sparrows (*Passer cinnamomeus*), both passerines using closed nests, in a montane farming area of Taiwan. We found that Asian house martins constructed dome‐shaped nests in human houses that provided additional nest sites for russet sparrows, secondary cavity nesters with greatly declining populations in Taiwan. Russet sparrows that used house martin nests had reproductive success comparable to those that used artificial nest boxes. However, Asian house martins avoided reclaiming sparrow‐used nests, which reduced their available nest sites. Interestingly, our results imply that man‐made structures may be used as a conservation tool to improve the breeding of the endangered russet sparrows via this facilitative interaction.

## Graphical abstract

We reported a facilitative species interaction between Asian house martins and russet sparrows in a montane farming area of Taiwan. We found that Asian house martins constructed dome‐shaped nests in human buildings that provided additional nest sites for russet sparrows, secondary cavity nesters with greatly declining populations in Taiwan. Russet sparrows that used house martin nests had reproductive success comparable to ones that used nest boxes resembling their cavity nests.

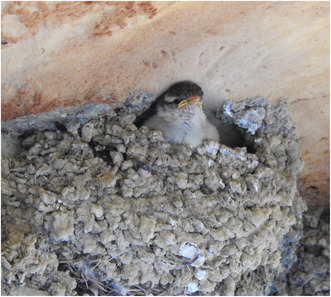

## 1 INTRODUCTION

Human‐modified habitats, such as urban areas and farmlands, tend to favor some species (e.g., synanthropic species) over others, thereby altering species distribution, community compositions, and biodiversity (Chace & Walsh, 2006; Wretenberg et al., 2010). However, complex species interactions may modify the outcome. While the role of antagonistic interactions (e.g., predation, competition) in mediating anthropogenic influences on animal communities is well recognized, facilitative interaction—an interaction in which one species enhances the growth, survival, or reproduction of a second species (Bronstein, 2009)—is relatively under‐studied until the recent decades (Stachowicz, 2001; Wright et al., 2017). For example, Hernández‐Brito et al. (2020) demonstrated that facilitative nesting association between an invasive (facilitated) and a native (facilitator) bird might have allowed for the spread of the invasive bird into the rural environment.

Cavity‐nesting birds are often considered adaptive to human‐modified habitats because man‐made structures provide them with ample nesting sites (Tomasevic & Marzluff, 2017). Even though bird nests associated with man‐made structures may have the benefits of protection against bad weather (Mainwaring, 2015) and brood parasitism (Liang et al., 2013), they are still vulnerable to predation (Wang & Hung, 2019) and usurpation (Bailey et al., 2020; Leasure et al., 2010). For example, Leasure et al. (2010) found that cliff swallows (*Petrochelidon pyrrhonota*) that build nests under bridges suffered nest usurpation by house sparrows (*Passer domesticus*) and had reduced breeding success. Given the rapid and large‐scale conversion of natural habitats to anthropogenic habitats, there is an urgent need to understand how cavity‐nesting species interact in human‐modified habitats and its consequences on population dynamics and community compositions.

Secondary cavity nesters—species that do not generate their own cavities but use those made by other species or formed naturally—benefit from primary cavity nesters. Such positive nesting association is a form of facilitative interaction. In human‐modified habitats, the nesting association between primary and secondary cavity nesters may become highly dynamic. On the one hand, by providing man‐made structures as nesting sites for primary cavity nesters, the presence of humans may enhance the facilitative interaction between primary and secondary cavity nesters. On the other hand, humans may weaken the facilitative interaction if secondary cavity nesters can directly use man‐made structures as nests and consequently reduce their dependence on primary cavity nesters (Tomasevic & Marzluff, 2017). Birds that build dome‐shaped nests using man‐made structures may play the same ecological role as do primary cavity nesters, providing nests for secondary cavity nesters (Leasure et al., 2010).

While common in the Himalayas region and southern China, the russet sparrow (*Passer cinnamomeus*) is listed as “endangered” by Taiwan's Wildlife Conservation Act due to its island‐wide population decline in recent years (Lin et al., 2016). Despite that we know little about their breeding ecology, they are documented secondary cavity nesters that use a variety of nest sites, including tree holes, cavities in rocks, man‐made structures, and artificial nest boxes (Yang et al., 2012; Yang et al., 2020; Ye et al., 2019). Here, we reported field evidence based on a 3‐year survey in a montane area of Taiwan that Asian house martins (*Delichon dasypus*), a synanthropic species building dome‐shaped nests in human houses, provided nests to russet sparrows. This is the first record demonstrating a facilitative interaction between russet sparrows and Asian house martins mediated by humans.

## 2 METHODS

### 2.1 Study site and species

**FIGURE 1:**
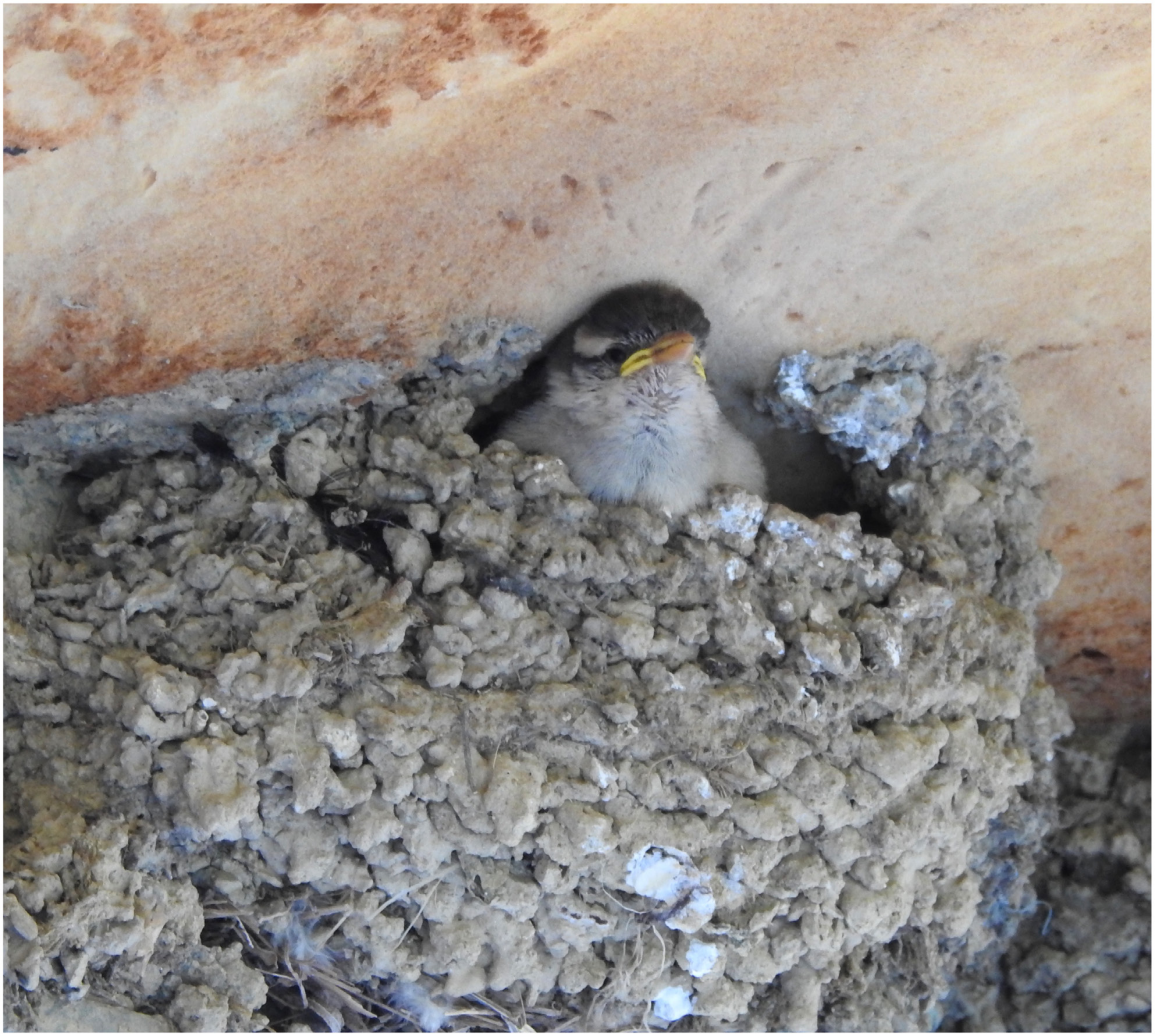
One russet sparrow chick with it head out of a dome‐shaped nest built by Asian house martins (photo by C.‐M. Hung)

This study was based on a 3‐year survey of another project focusing on the breeding ecology of Asian house martins, which was conducted from late March to early October (the breeding season of this bird) during 2019–2021 in Lishan, a montane farming area in Taiwan (24°19′36.2″ N, 121°18′24.6″ E) at an elevation of 1900 m. During the study periods, we found that russet sparrows used Asian house martin nests for breeding (Figure 1) from April to August every year. The landscape of the study area is a mosaic of large patches of orchards growing apples, pears, and peaches and small patches of secondary forests (Figure S1). The study site included two buildings ca. 200 m apart. There were about 100–140 Asian martin nests found at each building every year. Many of the nests were reused by Asian house martins over the years. Asian house martins build dome‐shaped nests under the eaves, and therefore, these nests are well protected from harsh weather. These cavity‐like martin nests may provide potential nest sites for secondary cavity nesters such as russet sparrows.

### 2.2 Breeding surveys

We checked the nests of Asian house martins in the mornings using an endoscope. We recorded the status of the nests (occupied by russet sparrows or Asian house martins) and the numbers and developmental stages of eggs and chicks in each brood. The survey was done at regular intervals in 2020 and 2021 (once every 3–4 days in 2020 and daily in 2021) and at irregular intervals in 2019. We quantified the breeding performance of Asian house martins using the following parameters: (1) egg number (EN)—the number of eggs laid; (2) hatchling number (HN)—the number of eggs hatched; (3) hatching success (HS)—the percentage of eggs hatched; (4) fledgling number (FN)—the number of hatchlings fledged; (5) fledging success (FS)—the percentage of hatchlings fledged; and (6) breeding success (BS)—the percentage of eggs fledged. We defined the developmental stage of “fledging” as: (1) one chick that was at least 12‐day old was found in the nest (Yang et al., 2012) and (2) an empty nest with no chick carcass nearby, suggesting the chicks had successfully fledged rather than being predated.

We also set up nest boxes that mimic cavities nests for russet sparrows at locations within 12 km from the study site at the elevation of 1500–2000 m during 2020–2021 for another project (Tsai, unpublished data). The survey of the nest‐box project was done at a relatively low frequency (3–4 times per month), making it difficult to assess fledging conditions. Therefore, from this data set, we only estimated three breeding parameters for russet sparrows: (1) egg number (EN), (2) hatchling number (HN), and (3) hatching success (HS).

### 2.3 Data analyses

First, we calculated the frequencies of Asian house martin nests used by russet sparrows and Asian house martins in 2020 and 2021, respectively. We then compared the reuse rate from 2020 to 2021 (the percentage of the 2020 nests that were reused in 2021 by russet sparrows and Asian house martins) to assess the strength of nesting association between these two species. The 2019 data were excluded from this analysis due to unclear nest ownership. Second, to assess whether the breeding of the russet sparrows using Asian house martin nests is generally successful, we calculated the median, 25–75th, and 5–95th percentiles of their EN, HN, HS, FN, FS, and BS. For this analysis, we pooled the data from 2019 to 2021 (*n* = 14). Finally, we compared the median, 25–75th, and 5–95th percentiles of EN, HN, and HS between russet sparrows that bred in nest boxes and martin nests. For this analysis, we pooled the data from 2020 to 2021 (*n* = 10 and 46 clutches in martin nests and nest boxes, respectively). All analyses were conducted in R v4.1.1 (R Core Team, 2021).

## 3 RESULTS

### 3.1 Asian house martin nests used by russet sparrows and Asian house martins

Four Asian house martin nests were used by russet sparrows in 2020 and 2021, respectively, resulting in ca. 1.6% of all martin nests (4/248 in 2020 and 4/239 in 2021) being used by russet sparrows. None of the four nests used by russet sparrows in 2020 were subsequently used by martins in 2021 (Table 1), suggesting that the martins might be avoiding nests that were used by russet sparrows in the previous year. This speculation was further supported by the high reuse rate of the 2020 nests in 2021 by the martins for those nests that were previously used by the martins (78%, 120/153) or unused (68%, 59/87; Table 1).

**TABLE 1:** Reuse rates of Asian house martin nests by russet sparrows and Asian house martins

Nest usage status In 2020	Used by martins in 2021	Not used by martins in 2021	Nest reuse rate by martins in 2021
Used by sparrows	0	4	0% (0/4)
Used by martins	120	33	78% (120/153)
Used by neither	59	28	68% (59/87)

*Note*: Of the 248 Asian house martin nests surveyed in 2020, 244 remained available for reuse in 2021. Nest reuse rates in 2021 were therefore calculated as the percentage of the 2020 nests reused by Asian house martins in 2021.

### 3.2 Breeding performance of russet sparrows in Asian house martin nests

**FIGURE 2:**
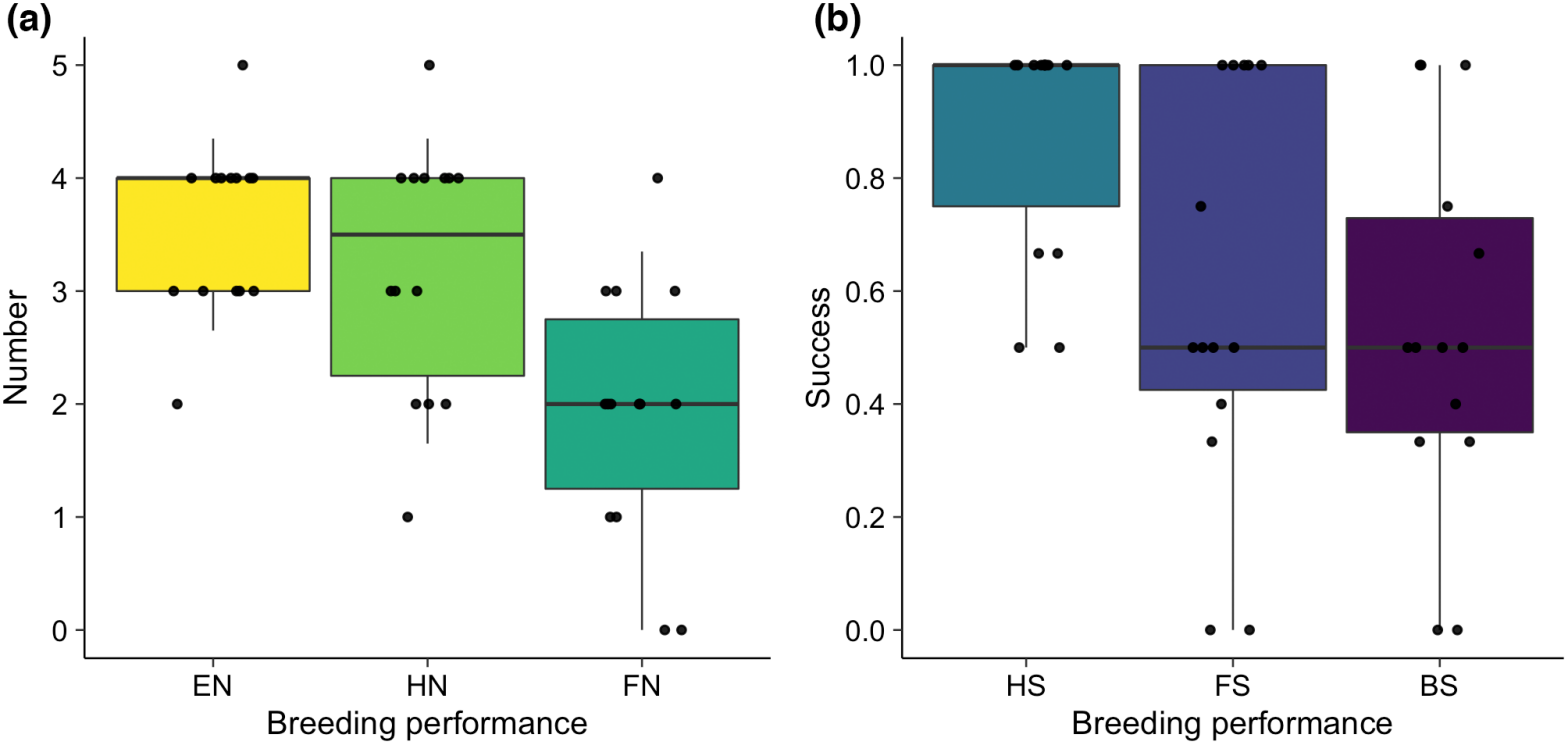
Breeding performance of russet sparrows using Asian house martin nests. Six breeding parameters were estimated: Egg number (EN), hatchling number (HN), fledgling number (FN), hatching success (HS), fledgling success (FS), and breeding success (BS). The first three parameters were based on counts (a) and the last three on proportions (b). The solid horizontal lines denote the median, and the boxes and whiskers denote the 25–75th and 5–95th percentiles, respectively. Each circle denotes a sparrow clutch, with a small horizontal jittering added for visual clarity.

**FIGURE 3:**
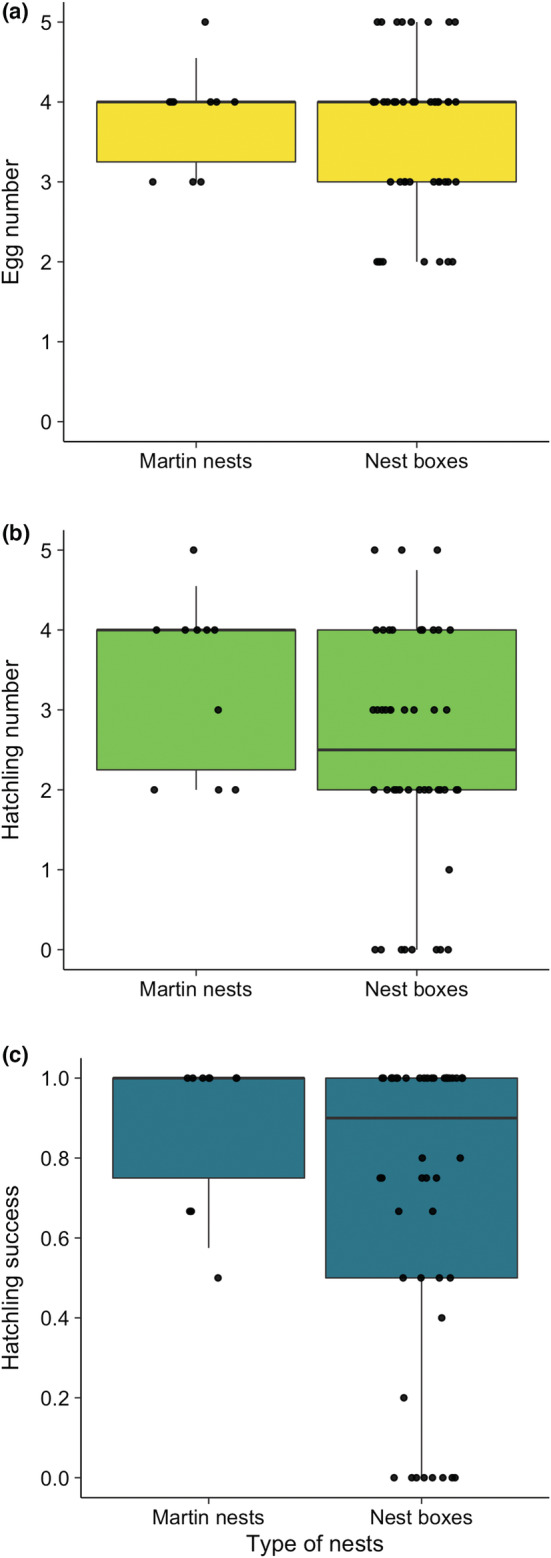
Comparison of breeding performance of russet sparrows using artificial nest boxes and Asian house martin nests. Three breeding parameters were estimated (a–c): Egg number (EN), hatchling number (HN), and hatching success (HS). The solid horizontal lines denote the median, and the boxes and whiskers denote the 25–75th and 5–95th percentiles, respectively. Each circle denotes a sparrow clutch, with a small horizontal jittering added for visual clarity.

There were 14 russet sparrow clutches during the entire study period (i.e., 4, 5, and 5 clutches in 2019, 2020, and 2021, respectively; more than one clutches could be from a given nest). Despite the slightly lower breeding performance in 2019 compared with 2020–2021 (Table S1), the russet sparrows that bred in the martin nests were generally successful, having positive median values for all six breeding parameters (Figure 2). Furthermore, the sparrows that used martin nests had breeding performance similar to those that used artificial nest boxes at nearby sites, as evidenced by the overlapped 25–75th and 5–95th percentiles of breeding performance between the two groups (Figure 3). These results indicate that the martins facilitated the sparrows by providing suitable nests that are of quality similar to or slightly better than nest boxes.

### 3.3 Reuse of Asian house martin nests by russet sparrows

Three of the 14 (21%) martin nests used by the sparrows were reused within or across years. Specifically, two nests were used twice by the sparrows within the same year (one in 2020 and one in 2021). A third nest was also used by the sparrows twice, first in 2019 and again in 2020.

## 4 DISCUSSION

We showed that Asian house martins—a synanthropic species—facilitated russet sparrows, an endangered species in Taiwan, by providing them with suitable nests. Specifically, the russet sparrows using the martin nests generally had positive values for breeding parameters, indicating successful reproduction. On the other hand, Asian house martins avoided the nests once they were used by russet sparrows, suggesting the interaction may reduce the breeding chances of Asian house martins.

Primary cavity nesters have been regarded as keystone species that constitute a nest web, which is similar to a food web but describes interactions between nesting species (Bednarz et al., 2004; Martin et al., 2004). Man‐made structures are well known to provide nesting sites to synanthropic species such as house sparrows and barn swallows (*Hirundo rustica*; Mainwaring, 2015; Wang et al., 2021). Here, we propose that Asian house martins, by building dome‐shaped nests in houses, can function as a keystone species similar to primary cavity nesters in a nest web, because they provide nests to secondary cavity nesters. In fact, Asian house martin nests not only served as suitable nests for russet sparrows, but they also allow the sparrows to breed in close proximity to humans. That is, Asian house martins may promote the adaptation of russet sparrows to human‐modified habitats.

The breeding performance of russet sparrows in 2019 was lower than in 2020 and 2021 (Table S1). This difference could be due to a low and irregular survey frequency in 2019, which might lead to underestimated breeding success. By contrast, the survey frequencies in 2020 and 2021 were higher and more regular, contributing to their higher and likely more accurate estimates of breeding performance. This highlights the importance of survey design in avian breeding studies.

**FIGURE 4:**
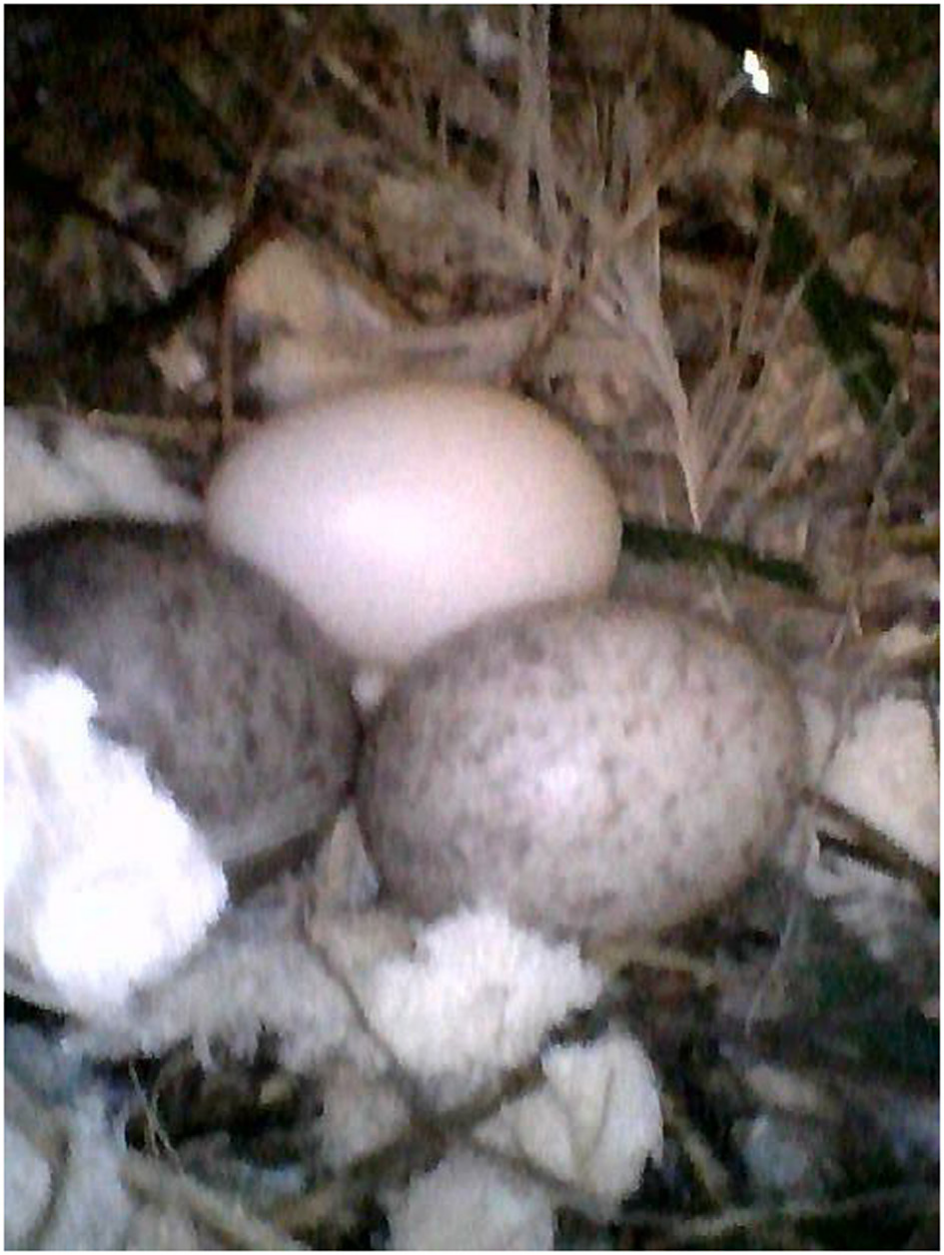
Two russet sparrow eggs (the spotty eggs) and one Asian house martin egg (the nonspotty egg) in the same nest (photo by J.‐S. Wang).

Although the four nests used by the sparrows in 2021 were built and used by the martins in 2020, we did not directly observe that the sparrows usurped nests from the martins. Surprisingly, we found one case where russet sparrows might engage in brood parasitism: In 2019, two sparrow eggs and one martin egg were found in the same martin nest (Figure 4). However, the fates of these eggs were uncertain and whether brood parasitism occurs between the russet sparrows and Asian house martins remains to be confirmed. Experimental studies showed that russet sparrows could recognize and reject chicks of cuckoos or other parasites from their nests (Huo et al., 2018), but common house martins (*Delichon urbicum*)—sister to Asian house martins—did not reject alien eggs from their nests (Liang et al., 2013). These studies imply that russet sparrows may have a better defense against brood parasitism than Asian house martins.

McNeil and Clark (1977) reported that house sparrows usurped the nests of common house martins, enlarged nest entrances, and introduced massive lining into the nests. We found that russet sparrows also introduced more and different kinds of linings (J.‐C. Guo, personal observation) into Asian house martin nests but did not change nest structure. Removing and reconstructing the linings of nests would be energy costly, which might explain why Asian house martins did not reclaim the nests after russet sparrows used them. Leasure et al. (2010) reported a similar phenomenon in cliff swallows and house sparrows; they argue that cliff swallows have worse breeding performance in nesting colonies with more house sparrows because house sparrows defend larger nesting ranges that prevent cliff swallows from breeding around them. However, in our study the nests used by russet sparrows were mostly in one corner of the martin colonies (data not shown), therefore the impact of sparrows is likely limited to a small portion of the Asian house martin colonies. Overall, our results suggest that russet sparrows may not directly drive martins out of the nests, but the fitness of martins may still decrease due to a loss of available nests.

The facilitative interaction among avian species via a nest web operating in human‐modified habitats, as reported in this study, may provide an opportunity for designing novel conservation tools for the russet sparrows and other endangered birds.

## AUTHOR CONTRIBUTIONS

**Jing‐Chia Guo:** Conceptualization (equal); data curation (lead); formal analysis (lead); writing – original draft (lead). **Jo‐Szu Tsai:** Data curation (equal); resources (equal); writing – review and editing (supporting). **Jhih‐Syuan Wang:** Data curation (equal). **Ya‐Wen Lin:** Data curation (equal). **Pei‐Jen L Shaner:** Conceptualization (equal); formal analysis (supporting); funding acquisition (equal); resources (equal); writing – original draft (equal). **Chih‐Ming Hung:** Conceptualization (equal); funding acquisition (lead); resources (lead); project administration (lead); writing ‐ original draft (lead).

## CONFLICT OF INTEREST

There is no conflict of interest to declare.

## Supporting information


Appendix S1

## Data Availability

The data that support the findings of this study is deposited in the Dryad repository (https://doi.org/10.5061/dryad.2bvq83bt7).
