# Supplementary material for: The role of a synanthropic bird in the nest niche expansion of a secondary cavity nester to man‐made structures
Source: Ecol Evol. 2022 Aug 4;12(8):e9188. doi: 10.1002/ece3.9188 (PMC9353230; doi:10.1002/ece3.9188)
Supplement: Supplementary file 1 — Appendix S1 [file ECE3-12-e9188-s001.docx]

**Title: The role of a synanthropic bird in the nest niche expansion of a secondary cavity-nester to man-made structures**

Jing-Chia Guo^1^, Jo-Szu Tsai^2^, Jhih-Syuan Wang^3^, Ya-Wen Lin^2^, Pei-Jen Lee Shaner^1^* & Chih-Ming Hung^3^*

^1^ Department of Life Sciences, National Taiwan Normal University, Taipei, Taiwan

^2^ Department of Biological Resources, National Chiayi University, Chiayi, Taiwan

^3^ Biodiversity Research Center, Academia Sinica, Taipei, Taiwan

*Corresponding authors:

[pshaner@ntnu.edu.tw](mailto:pshaner@ntnu.edu.tw) (P.-J. Shaner); [cmhung@gate.sinica.edu.tw](mailto:cmhung@gate.sinica.edu.tw) (C.-M. Hung)

**Supplementary Information:**

**Table S1**

**Figure S1**

**Table S1.** Breeding performance of the russet sparrows using Asian house martin nests across 2019-2021. The numbers of clutches are 4, 5, and 5 for 2019, 2020, and 2021, respectively. The median value with the range (minimum-maximum) for each breeding performance parameter was presented.

| Breeding performance parameter | 2019  (n=4) | 2020  (n=5) | 2021  (n=5) |
| --- | --- | --- | --- |
| Egg number (EN) | 3 (2-4) | 4 (3-4) | 4 (3-5) |
| Hatchling number (HN) | 3 (1-4) | 4 (2-4) | 4 (2-5) |
| Hatching success (HS) | 1 (0.5-1) | 1 (0.5-1) | 1 (0.7-1) |
| Fledgling number (FN) | 0.5 (0-3) | 2 (1-3) | 2 (2-4) |
| Fledging success (FS) | 0.2 (0-1) | 0.5 (0.5-1) | 1 (0.4-1) |
| Breeding success (BS) | 0.2 (0-1) | 0.5 (0.3-0.8) | 0.7 (0.4-1) |


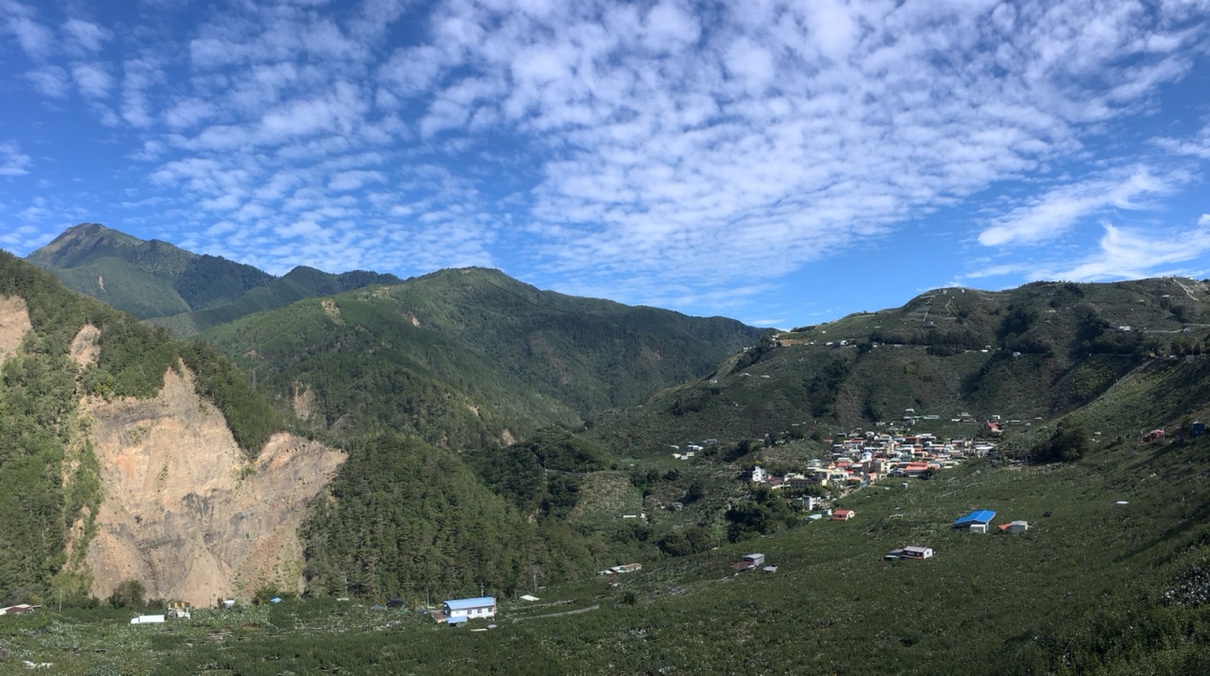


**Figure S1.** The landscape of the study area is a mosaic of large patches of orchards growing temperate fruits and small patches of secondary forests (Photo by J.-C. Guo).
